# Move to Remember: The Role of Physical Activity and Exercise in Preserving and Enhancing Cognitive Function in Aging—A Narrative Review

**DOI:** 10.3390/geriatrics10060143

**Published:** 2025-11-05

**Authors:** Alexandra Martín-Rodríguez, Athanasios A. Dalamitros, Rubén Madrigal-Cerezo, Paula Sánchez-Conde, Vicente Javier Clemente Suárez, José Francisco Tornero Aguilera

**Affiliations:** 1 Faculty of Medicine, Health and Sports, Universidad Europea de Madrid, Villaviciosa de Odon, 28670 Madrid, Spain; vctxente@yahoo.es; 2 Faculty of Health Sciences, UNIE University, 28015 Madrid, Spain; 3 Laboratory of Evaluation of Human Biological Performance, Department of Physical Education and Sport Science, Aristotle University of Thessaloniki, 57001 Thessaloniki, Greece; dalammi@phed.auth.gr; 4 Faculty of Education Sciences, UNIE University, 28015 Madrid, Spain; ruben.madrigal@universidadunie.com; 5 Faculty of Biomedical and Health Sciences, Department of Psychology, Villaviciosa de Odon, 28670 Madrid, Spain; paula.sanchez@universidadeuropea.es; 6 Grupo de Investigación en Cultura, Educación y Sociedad, Universidad de la Costa, Barranquilla 080002, Colombia; 7 Graduate School of Business, Universidad ESAN, Alonso de Molina 1652, Santiago de Surco, Lima 15023, Peru; doctorneroaguilera@gmail.com

**Keywords:** physical activity, aging, cognition, neuroplasticity, BDNF, glymphatic system, myokines, APOE ε4, exercise intervention

## Abstract

**Background/Objectives**: The global aging population faces rising rates of cognitive decline and neurodegenerative disorders. This review explores how physical exercise influences brain health in aging, focusing on mechanisms, moderators, and personalized strategies to enhance cognitive resilience. **Methods**: A narrative review methodology was applied. Literature published between 2015 and 2025 was retrieved from PubMed, Scopus, and Web of Science using keywords and MeSH terms related to exercise, cognition, neuroplasticity, aging, and dementia. Inclusion criteria targeted peer-reviewed original studies in humans aged ≥60 years or aged animal models, examining exercise-induced cognitive or neurobiological outcomes. **Results**: Evidence shows that regular physical activity improves executive function, memory, and processing speed in older adults, including those with mild impairment or genetic risk (e.g., APOE ε4). Exercise promotes neuroplasticity through increased levels of BDNF, IGF-1, and irisin, and enhances brain structure and functional connectivity. It also improves glymphatic clearance and modulates inflammation and circadian rhythms. Myokines act as messengers between muscle and brain, mediating many of these effects. Cognitive benefits vary with exercise type, intensity, and individual factors such as age, sex, chronotype, and baseline fitness. Combined interventions—physical, cognitive, nutritional—show synergistic outcomes. Digital tools (e.g., tele-exercise, gamification) offer scalable ways to sustain engagement and cognitive function. **Conclusions**: Physical exercise is a key non-pharmacological strategy to support cognitive health in aging. It acts through diverse systemic, molecular, and neurofunctional pathways. Tailored exercise programs, informed by individual profiles and emerging technologies, hold promise for delaying or preventing cognitive decline.

## 1. Introduction

The demographic shift toward an aging global population presents one of the most pressing public health challenges of the 21st century. By 2050, the proportion of individuals aged 60 years and older is expected to double, reaching over two billion worldwide [1]. In addition to physical activity, other lifestyle factors such as balanced nutrition, adequate sleep, and sustained social engagement have also been linked to cognitive health in aging. These elements are increasingly recognized as part of a multidomain framework for dementia prevention. Within this broader context, exercise represents a central and modifiable pillar that interacts with and complements these factors, reinforcing its role as a key target for promoting healthy brain aging [1,2,3,4,5,6]. An important conceptual distinction should be noted between physical activity and exercise. Physical activity refers to any bodily movement produced by skeletal muscles that results in energy expenditure, and it can encompass occupational, sports, conditioning, household, or other daily activities. Exercise, in contrast, is a subset of physical activity that is planned, structured, and repetitive, with the specific objective of improving or maintaining physical fitness. Recognizing these definitions is relevant for the present review, as they provide a clearer framework for interpreting the diverse findings across studies on aging, cognition, and brain health [6,7].

With increased longevity comes a higher prevalence of age-associated cognitive impairments, ranging from mild cognitive decline to neurodegenerative disorders such as Alzheimer’s disease and related dementias [2]. These conditions significantly compromise autonomy, daily functioning, and quality of life, while imposing substantial economic burdens on healthcare systems [3]. Importantly, cognitive decline does not occur uniformly; rather, it reflects a complex interplay between biological aging processes, environmental exposures, and modifiable lifestyle factors [4]. In this context, identifying non-pharmacological strategies to maintain or enhance cognitive health in older adults has become a research and clinical imperative. Among these strategies, physical exercise has garnered substantial attention for its neuroprotective potential and broad accessibility across aging populations [5].

A growing body of empirical evidence supports the role of physical exercise as a powerful modulator of cognitive function in aging. Both cross-sectional and longitudinal studies have demonstrated that physically active older adults exhibit superior performance in domains such as memory, attention, executive functioning, and processing speed when compared to physically inactive peers [6,7]. Randomized controlled trials further confirm that structured exercise interventions, particularly aerobic and multicomponent programs, can elicit significant improvements in global cognition and domain-specific outcomes in both cognitively healthy and mildly impaired older individuals [8]. Neuroimaging studies provide converging support, revealing exercise-induced changes in brain structure and function, including increased hippocampal volume, enhanced white matter integrity, and improved functional connectivity in key cognitive networks [9,10]. These cognitive benefits are thought to be mediated by several interrelated mechanisms, including increased cerebral blood flow, neurogenesis, synaptic plasticity, angiogenesis, and upregulation of neurotrophic factors such as brain-derived neurotrophic factor (BDNF) and insulin-like growth factor-1 (IGF-1) [11,12]. However, the magnitude and sustainability of these effects appear to depend on multiple variables, including the type, intensity, frequency, and duration of physical activity, as well as the individual’s age, sex, baseline fitness, and cognitive status [13].

Despite the wealth of studies supporting the cognitive benefits of exercise, the current literature often remains fragmented, with limited integration across the diverse biological, behavioral, and technological domains involved in brain aging. Many studies focus on isolated mechanisms, such as neurotrophic signaling or hippocampal volume, without linking these findings to functional outcomes or population-specific factors [13,14,15]. Furthermore, there is insufficient clarity regarding the optimal exercise modalities, intensities, and durations necessary to maximize cognitive benefit in distinct subgroups of older adults, including those with different genetic risk profiles, sex differences, circadian typologies, or comorbidities [11,12,13]. The emergence of novel topics such as the glymphatic system, the myokine-mediated muscle–brain axis, and the use of digital tools like tele-exercise and cognitive-motor gamification highlights the complexity of the field and the need for an integrative framework. Therefore, a comprehensive synthesis that spans molecular, systemic, and behavioral levels is urgently needed to guide precision-based cognitive interventions in aging populations. This narrative review addresses that gap by integrating current evidence on how various forms of physical activity influence brain structure and function, with the aim of identifying mechanisms, moderators, and practical applications for personalized cognitive health promotion.

### 1.1. Methods

#### 1.1.1. Design

This review was conducted as a narrative review, which is particularly suited for integrating heterogeneous findings from human and animal studies on exercise, cognition, and brain aging. Narrative reviews are particularly appropriate when addressing complex, interdisciplinary phenomena that cannot be adequately captured through quantitative aggregation alone [14,16,17]. Given the multifactorial nature of cognitive aging encompassing neural, metabolic, psychological, and behavioral domains, this method enables the contextual integration of molecular mechanisms, neuroimaging findings, and population-level outcomes. Prior studies have demonstrated the value of this approach in health sciences, where interactions among nutrition, brain function, microbiota, and physical activity require interpretive synthesis rather than strict meta-analytical criteria [18,19,20].

#### 1.1.2. Search Strategy

Boolean operators were applied to combine concepts related to exercise, aging, and cognition. The main search path used was:

(“physical activity” OR “exercise”) AND (“cognitive function” OR “memory” OR “executive function”) AND (“aging” OR “older adults” OR “elderly”) AND (“neuroplasticity” OR “brain health”).

Additional MeSH term combinations included:

“exercise” AND “neuroplasticity” AND “aging”

“aerobic training” OR “resistance training” AND “cognitive decline”

“physical activity” AND “dementia prevention”

Filters were applied for: humans or relevant animal models, age ≥ 60 years, peer-reviewed original research articles, and publication date between 2015 and 2025.

#### 1.1.3. Eligibility Criteria

In total, approximately 250 original studies that met these criteria were included for detailed analysis in the present review. Inclusion criteria following previous research [20] were defined as follows:

1. Original, peer-reviewed journal articles published in English between January 2015 and May 2025.
2. Human studies involving participants aged ≥60 years or animal models relevant to cognitive aging.
3. Investigation of at least one of the following: cognitive outcomes of exercise interventions; molecular mediators of neuroplasticity; exercise-induced brain structural or functional changes; or interactions between physical activity and cognitive risk factors.

Exclusion criteria were conference abstracts, preprints, dissertations, and grey literature. Review articles were used for conceptual background but excluded from the analytical synthesis. Human studies were prioritized in the narrative, while animal studies were considered to support mechanistic interpretation.

#### 1.1.4. Data Extraction and Synthesis

Data extraction was conducted manually using a structured coding scheme. For each study, we collected information on the study population (e.g., healthy older adults, individuals with mild cognitive impairment, APOE ε4 carriers, or relevant animal models), the type of intervention (aerobic, resistance, high-intensity interval training, or multimodal), the main characteristics of the intervention (duration, frequency, and intensity), and the cognitive and neurobiological outcomes reported. The included studies were then thematically clustered into six domains: (1) cognitive effects of physical activity across domains, (2) exercise-induced neuroplasticity and neurotrophic pathways, (3) the muscle–brain axis, (4) network-level and glymphatic adaptations, (5) moderators of exercise efficacy such as genetics, sex, and circadian rhythms, and (6) translational and personalized strategies for cognitive health promotion. This organization allowed us to synthesize findings in a coherent framework, highlighting both mechanistic insights and their practical implications for cognitive aging.

#### 1.1.5. Figure Design and Source Attribution

All figures were created by the authors using the web-based software, BioRender.com (accessed on 20 June 2025), for conceptual illustration purposes. The authors have reviewed and edited all content and take full responsibility for the final version. No copyrighted or previously published materials were used.

## 2. Cognitive Aging as a Global Health Challenge

Life expectancy has increased over the last decades [21,22], except for the years when the global pandemic related to COVID-19 had a major impact on the number of deaths, leading to a decline in life expectancy, which has subsequently been restored. All of this, along with multiple advances in the field of medicine and science, has led to the analysis of health-related areas, studying its preservation and treatment in the event that it is impaired, among others [23,24].

While the benefits of ageing, linked to longer life expectancy of the population, may be notable for individuals themselves or for society as a whole, it also poses challenges, including health challenges, that need to be addressed [25]. Such challenges are even more attractive and necessary as the world population is expected to grow (8 billion people is the current estimate) and increase by approximately 1 billion people in the next twelve years [26]. Nevertheless, the rise in numbers also means, for example, that the number of people living with dementia is estimated to grow from 55 million to 139 million by 2050 [27,28]. One of these ongoing research areas is related to cognitive ageing, which can be defined as a heterogeneous process [29] whereby some areas deteriorate. This impairment can be related to knowledge and to certain capacities, such as attention, audiovisual perception, or executive functions [30].

Therefore, it is also necessary to distinguish between normal cognitive ageing and pathological ageing, the first being understood, as where certain capacities decrease over time, such as memory, processing speed or conceptual reasoning. Following these authors, other cognitive abilities such as vocabulary may improve or resist ageing. From this perspective, cognitive aging can be characterised by a notable degree of heterogeneity [31]. When we refer to pathological cognitive ageing [32], we are describing processes that go beyond the features of normal cognitive ageing and are triggered by neurodegenerative or vascular mechanisms among others [33].

From a different perspective, preserving and improving cognitive functions within ageing can help authorities and society in a positive health, economic and political sense. Health benefits will be discussed later, but it is convenient to briefly outline that slowing down aging generates complementary benefits in terms of health and longevity, leading to a vicious circle where slowing down aging produces greater longevity [34]. From a political perspective, the cohort study conducted among the Chinese population (Longitudinal Study of Health and Retirement) and the English population (English Longitudinal Study of Aging) indicated that education was beneficial for cognitive resilience in mid-to-late life, inclusive education appears to be an international priority in promoting healthy cognitive aging, and that formal education can influence cognitive performance throughout life [35].

Thus, in the following paragraphs, we will focus on exploring how physical exercise has an impact and can sometimes be an ally in the onset of cognitive ageing in the adult population.

## 3. Exercise-Induced Neuroplasticity: Molecular Foundations

In this section, we will discuss the role played by neuroplasticity and the influence that physical exercise has on the process of cognitive ageing. In this sense, we refer to neuroplasticity as the biological capacity for intrinsically active adaptation of maturation and change in the structure and functioning of the central nervous system (CNS) after an injury [36]. It is not only the injury factor that triggers neuroplasticity, as we also refer to it as the brain’s ability to reorganise and modify neuronal connections in response to environmental stimuli, disease processes, learning and experience, in addition to the aforementioned injuries. We will now focus on how exercise can be an enabling factor in the process of neuroplasticity and its connection to ageing [37].

Research on mice has been developed on the impact of aerobic and high-intensity exercise. While improvements in spatial learning skills, recognition memory, neurogenesis and increased synaptic plasticity were found by implementing aerobic exercise in mice for 8 weeks performing 5 sessions per week and increasing the intensity by 5% per week [38] no changes in hippocampal neuroplasticity were observed in young adult mice during high-intensity physical training, although an increase in antioxidant defences for the prevention of brain damage was observed during aerobic exercise [39]. Previous studies have reported upregulation of several genes involved in synaptic plasticity and transcriptional regulation, along with reductions in anxiety and improvements in learning behavior. These findings suggest that physical activity may play a supportive role in promoting cognitive resilience and could contribute to lowering the risk of cognitive impairment and neurodegenerative disorders [40]. Finally, research conducted in non-transgenic mice on neuroprotective mechanisms of exercise in a postmenopausal animal model revealed that, after a three-month programme of voluntary exercise on a treadmill, exercise protected the mice against deleterious behaviours and normalised learning and memory. Also, that ovariotomy alters CREB (Cyclic AMP Response Element Binding Protein) activation through exercise from analyses of key markers of antioxidant signalling paths and neuroplasticity, and that BDNF (Brain-Derived Neurotropic Factor) plays a key role in neuroprotection [41]. In both animals and humans, exercise has been shown to reduce anxiety and improve cognition, without deepening its typology, but it is true that vigorous exercise can enhance neuroplasticity and stress resistance through adaptive cellular responses, including positive regulation of neurotrophic factor signalling, autophagy, DNA repair, stabilisation of calcium homeostasis, suppression of inflammation and oxidative stress or stimulation of mitochondrial biogenesis as highlighted for our purposes here [42].

Lactate has been another chemical compound analysed after high intensity exercise, which has also been linked to increased peripheral Brain Derived Neurotrophic Factor (BDNF) levels, that lactate infusion at rest is able to elevate peripheral and central BDNF levels and that lactate has a complex function within brain metabolism, as well as that this type of exercise can increase BDNF itself, cathepsin B and irisin [43]. This latter study was carried out in a review between animal and human studies. Encouraging physical exercise as an enabler of short- or long-term neuroplastic changes in the brain was also analysed. Thus, the connections that are activated in the process of late long-term synaptic potentiation (LTP) increase the transcription of growth and survival stimulating factors such as BDNF. In this regard, we aimed to generate a framework that structures the relevant information on exerkines involved in the regulation of early and late stages of LTP in the human brain to clarify the role of exerkines’ release on the enhancement of the LTP process induced by physical exercise. The role of BDNF, myokines, cytokines, metabolites, hormones and neuropeptides released during physical exercise with the potential to alter LTP-related pathways was studied. It was found that intense cardio and endurance exercise temporarily increased circulating BDNF, with sessions lasting more than 30 min inducing higher increases in circulating BDNF than shorter sessions [44]. Regarding chronic exercise, it is noted that further research is needed to establish positive or negative guidelines in this regard, both in healthy adults and in those who are not. There is also a lack of evidence on the effect of exercise-induced increases in GABA concentrations in cortical neurons, measured with 1 H-MRS, which may have GABAergic neurotransmission. Of the 16 exerquines analyzed, BDNF, irisin, and pro-inflammatory cytokines provided evidence suggesting that physical exercise at surrounding exerquine levels was associated with the enhancement or deterioration of LTP activity. Moreover, was found that EF-induced elevation in IGF-1, BHB, lactate, and irisin activated the transcription of one of the exerquines with a known facilitating effect on EF-induced LTP (BDNF or irisin). Lastly, the release of exerquines during and after great and chronic resistance exercise is limited compared to cardiovascular exercise. Furthermore, it is expected that the release of exergines requires a certain intensity of exercise before protein synthesis is activated. It should be noted, however, that higher intensity is not always better. Factors such as overtraining must be taken into account to avoid adverse effects. This latest article concludes that the effect of physical exercise is beneficial for cognitive function [45].

Adults have also been studied, with strength training being identified as a cognitive flexibility enhancer [46] that the introduction of new environments and tasks can be a key driver of exercise-induced neuroplasticity [47] focused on addressing the immediate and ultimate mechanisms underlying age-related brain atrophy and the influence of lifestyle changes on the course of healthy and pathological aging. Referring to physically inactive healthy adults, more specifically, it is stated that aerobic and combined exercise performed from The Project Movement Protocol led to subsequent studies where it was established that a 12-week aerobic exercise program improved attention-speed and executive function in healthy middle-aged adults. Its combination with computers-based cognitive training showed no added benefits [48]. Another study, unrelated to the aforementioned one, pointed out that greater cardiovascular fitness can contribute to several improvements in cognition, all of which have in common the upward regulation of neurotrophins (including BDNF), thereby generating better neuroplasticity [49].

In the case of specific diseases, each one must be analysed individually. Stroke and its relationship with physical exercise is positive as it improves motor recovery and helps reduce depression in people who have suffered a stroke, but there is no clear evidence that it is a positive sign in reversing subsequent cognitive impairment [50]. On the other hand, Alzheimer’s disease (AD) can be delayed in its onset and slowed in its progression through physical exercise. Exercise improves blood glucose levels and contributes to homeostatic regulation, where the circulating and central concentration of BDNF is elevated, which in turn improves neuronal function and synaptic plasticity, especially in the hippocampus [51]. Resistance training has also been demonstrated to be a significant factor in preventing AD [52]. ADHD has also been studied in regard to this section, concluding under his proposed exercises and analysis that irisin appears to be a prototypical molecule that can activate autophagy for therapeutic purposes [53].

Another type of exercise is high-intensity exercise. A meta-analysis was conducted to estimate the immediate effects of this type of exercise on BDNF levels in healthy young adults. The results showed that, compared to low-intensity exercise or no exercise, there was a positive effect on increasing BDNF levels (unrelated to the duration of the activity or the baseline cardiorespiratory fitness of healthy young adults). All of this supports the recommendation by doctors that high-intensity physical exercise is a promising and efficient approach to boosting BDNF levels [54]. In another study conducted under a High-Intensity Interval Training (HIIT) protocol, it was noted that there were no baseline changes in neurophysiological or molecular measures, although it is likely that this type of protocol prepares the motor system for great plasticity in physically inactive men. Furthermore, high levels of cardiorespiratory fitness were observed, which could result in higher levels of GABA and/or glutamate within M1 after performing acute exercise, despite no relationship being found between cardiorespiratory fitness and GABA or glutamate levels in M1 [55].

The difference between high- and low-intensity exercise has also been studied in adults who have suffered some type of injury or have some type of illness. A new study carried out through systematic review and analysis, shows evidence of this, as it was based on healthy young and older adults and patients with multiple sclerosis, stroke, or Parkinson’s disease. Finally, both types of exercise improved neuroplasticity, while also highlighting the need to individualise and regulate physical exercise stimulus in healthy young people, as it is not necessary in healthy older adults and neurological patients, thus alluding to the need for further comparative studies [56]. Exercise performed in aerobic and anaerobic phases has also been shown to increase insulin activity and transcription factor expression (IGF-1) in animals treated with corticosterone.

To conclude this section, it should be noted that research is needed to further investigate the effect of exercise on neuroplasticity in cognitive aging, whether in healthy adults or those with disease, of the different loads applicable in physical exercise, as well as the molecular connection of its induction [50,57,58]. Despite this, we have seen how the molecular factors BDNF, irisin, and IGF-1 are key factors in exercise-induced neuroplasticity and their action in cognitive aging.

## 4. The Muscle–Brain Axis: Myokines as Cognitive Mediators

### 4.1. Skeletal Muscle as an Endocrine Organ

In recent years, skeletal muscle has emerged as more than a motor structure: it is now conceptualized as a key endocrine organ. During physical exercise, contracting muscles release a variety of signaling molecules known as myokines, which enter the circulation and exert autocrine, paracrine, and endocrine effects [59]. This endocrine function creates a biochemical communication pathway between the musculoskeletal and central nervous systems, coined the muscle–brain axis, which has profound implications for cognitive function, particularly in the aging population [60,61,62].

This conceptual shift is supported by a growing body of molecular and translational studies showing that physical activity not only improves muscular fitness, but also initiates a cascade of neurobiological processes mediated by muscle-derived factors [63,64]. These systemic signals are thought to influence brain plasticity, inflammation, and even adult neurogenesis, all of which are critical in the context of neurodegeneration and cognitive decline.

### 4.2. Key Exercise-Induced Myokines and Their Cognitive Roles

Several myokines have been identified as crucial modulators of brain function (Table 1):

- Brain-Derived Neurotrophic Factor (BDNF): While BDNF is also synthesized within the brain, peripheral BDNF levels increase with exercise and correlate with improvements in memory and executive function. BDNF supports hippocampal neurogenesis, synaptic plasticity, and the maintenance of dendritic complexity—processes that are compromised in aging and Alzheimer’s disease [65].
- Irisin: This myokine is produced by the cleavage of the transmembrane protein FNDC5 in response to endurance training. Irisin crosses the blood–brain barrier and has been shown to elevate BDNF expression in the hippocampus, enhance synaptic strength, and improve learning and memory in rodent models [66,67].
- Cathepsin B: Identified in both human and animal studies, this lysosomal protease is released during aerobic activity and appears to contribute to hippocampal neurogenesis and spatial memory improvements. It may also play a role in the degradation of amyloid-beta aggregates [68].
- Interleukin-6 (IL-6): Although chronically elevated IL-6 is associated with neuroinflammation, acute increases during exercise serve an anti-inflammatory role, reducing the production of TNF-α and other pro-inflammatory cytokines. This shift contributes to a neuroprotective environment, especially important in the context of “inflammaging.” [69].
- Meteorin-like (METRNL): Recently discovered as a myokine upregulated by both cold exposure and exercise, METRNL exerts anti-inflammatory and neurotrophic effects by promoting M2 macrophage polarization and indirectly stimulating BDNF production in the brain. Its modulation of immune–brain crosstalk suggests relevance in neurodegenerative conditions [70].
- Fibroblast Growth Factor 21 (FGF21): Although primarily produced in the liver, skeletal muscle contraction can enhance FGF21 expression. This hormone-like factor crosses the BBB and may promote cognitive resilience by improving mitochondrial function, reducing oxidative stress, and upregulating autophagy pathways in neurons [71].

**Table 1:** Studies on Exercise-Induced Myokines and Cognitive Function.

Myokine	Key Findings	Study (Author, Year)	Model
**BDNF**	1. Peripheral BDNF increases after aerobic training and correlates with memory improvement in older adults. 2. 12-month aerobic intervention increased BDNF and delayed cognitive decline in older adults.	1. Erickson et al., 2011 [72]	1. Humans (older adults) 2. Humans (older adults)
**Irisin**	1. Irisin crosses BBB; enhances hippocampal BDNF expression and improves cognition in mice after exercise. 2. Forced treadmill running in mice increased FNDC5 expression and elevated hippocampal BDNF.	1. Wrann et al., 2013 [73] 2. Lourenco et al., 2019 [74]	1. Rodent models (mice) 2. Rodent models (mice)
**Cathepsin B**	1. Exercise-induced Cathepsin B correlates with spatial memory and neurogenesis in humans and mice. 2. Voluntary wheel running elevated cathepsin B and improved performance in object location memory task.	1. Moon et al., 2016 [68] 2. Trejo et al., 2008 [75]	1. Humans and mice 2. Rodent models (mice)
**IL-6**	1. Prolonged exercise-induced IL-6 reduces TNF-α levels and supports anti-inflammatory environment in CNS. 2. IL-6 knockout mice failed to exhibit exercise-induced neurogenesis, indicating its essential role.	1. Starkie et al., 2001 [76] 2. Tsuchida et al., 2022 [77]	1. Humans 2. Rodent models (mice)
**Meteorin-like (METRNL)**	1. Exercise elevates METRNL, promoting M2 macrophage polarization and BDNF-related neurotrophic effects. 2. METRNL deficiency impaired exercise-induced hippocampal neuroplasticity in animal models.	1. Rao et al., 2014 [78] 2. Hong et al., 2022 [79]	1. Mice 2. Rodent models (mice)
**FGF21**	1. Exercise increases FGF21 expression; improves neuronal mitochondrial function and cognitive outcomes in aged mice. 2. FGF21 administration improved synaptic plasticity and reduced tau pathology in aged mice.	1. Yang et al., 2021 [80] 2. Flippo et al., 2020 [81]	1. Mice 2. Rodent models (mice)

### 4.3. Mechanisms of Action and Blood–Brain Barrier Interaction

One of the key characteristics that makes myokines particularly relevant to brain health is their ability to either cross the blood–brain barrier (BBB) or modulate central processes indirectly through peripheral immune or endocrine interactions [82]. For example, irisin—first described by Fagundo et al. (2016) [83]—has been detected in cerebrospinal fluid (CSF) following endurance training, suggesting it reaches the central nervous system (CNS) and influences hippocampal BDNF expression [84]. Similarly, Cathepsin B, identified as an exercise-responsive myokine by Moon et al., has been shown to cross the BBB and enhance spatial memory and neurogenesis in mice [85].

Other myokines act indirectly by altering systemic inflammation or vascular health [86,87,88]. For instance, IL-6 released acutely during exercise exerts anti-inflammatory effects by stimulating the production of IL-10 and inhibiting TNF-α, a mechanism highlighted by Pedersen and Febbraio as part of the exercise-induced anti-inflammatory response. METRNL [60], a recently identified myokine, has also been implicated in promoting M2 macrophage polarization and anti-inflammatory cytokine production, thereby creating a peripheral immune environment conducive to CNS homeostasis [89].

At the intracellular level, myokines such as BDNF and irisin activate key signaling cascades involved in synaptic plasticity and neuronal survival. BDNF, for example, binds to the TrkB receptor, triggering downstream pathways including PI3K/Akt, MAPK/ERK, and CREB phosphorylation—mechanisms extensively reviewed by Lu et al. [90]. These signaling pathways not only support neuroplasticity and long-term potentiation but also promote mitochondrial biogenesis via PGC-1α activation [84].

Moreover, physical activity-induced myokines are increasingly associated with epigenetic modulation in the CNS. Exercise elevates histone acetylation and DNA demethylation patterns linked to memory-related gene expression, a process in which myokines like irisin and BDNF may play a mediating role [91,92,93].

Taken together, these findings indicate that myokines serve as molecular messengers bridging peripheral muscle activity with central nervous system adaptations, via both direct BBB interaction and indirect systemic modulation. Their influence on neuroplastic signaling, neuroinflammation, and epigenetic programming makes them key targets for interventions aiming to preserve cognitive function during aging.

### 4.4. Implications for Cognitive Aging and Intervention

The discovery of the muscle–brain axis has opened new avenues for interventions targeting cognitive decline in aging [82]. As the aging population faces an increased prevalence of neurodegenerative conditions such as Alzheimer’s disease and age-related cognitive impairment, physical activity has emerged as a potent non-pharmacological strategy to mitigate neural deterioration [94]. Specifically, exercise-induced myokines such as BDNF, irisin, and cathepsin B have been consistently associated with improvements in hippocampal volume, executive function, and episodic memory in older adults [72].

Tailoring physical exercise protocols—particularly those that optimize aerobic and resistance modalities known to stimulate myokine release may help clinicians design targeted interventions that enhance neuroplasticity and delay or prevent the onset of cognitive impairment [95]. For example, Maass et al. and Leger et al. reported that aerobic exercise programs of moderate-to-high intensity have been shown to elevate circulating irisin and BDNF levels, leading to improved cognitive outcomes in both clinical and non-clinical elderly populations [96,97].

Moreover, the concept of individual variability in myokine responsiveness is gaining traction. Genetic and epigenetic differences, metabolic health, and baseline fitness levels may all influence how an individual’s muscle tissue responds to exercise stimuli, thereby modulating the release and central action of myokines [98]. Understanding these interindividual factors could pave the way for personalized exercise prescriptions, especially in geriatric care settings where standard protocols may not be equally effective for all.

In addition, recent translational studies suggest that multi-domain interventions—combining physical activity with cognitive training, nutritional support, and social engagement can synergistically enhance cognitive resilience [99,100,101]. Within these frameworks, myokine-mediated pathways may serve as key biological mediators underlying the benefits of physical components.

Altogether, integrating knowledge of the muscle–brain axis into clinical and public health strategies offers a promising and accessible avenue to promote healthy cognitive aging. Continued research into myokine signaling, optimal exercise parameters, and interindividual differences will be essential to fully harness the neuroprotective potential of physical activity across the lifespan.

## 5. Stimulation of the Glymphatic System Through Physical Activity

In recent years, growing attention has been directed toward the glymphatic system as a novel target for brain health interventions. This perivascular network, responsible for clearing neurotoxic waste from the central nervous system, has emerged as an important mechanism associated with brain health and may contribute to reducing the risk of neurodegenerative diseases. While its function is primarily studied in relation to sleep, emerging evidence suggests that physical activity also plays a significant role in modulating glymphatic efficiency. The following subsections explore how exercise influences this clearance system, from structural and molecular mechanisms to its implications for aging and brain pathology [102,103].

### 5.1. The Glymphatic System: An Overview

The glymphatic system, a brain-wide perivascular network, plays a crucial role in maintaining neural homeostasis by facilitating the clearance of interstitial waste products—including amyloid-beta (Aβ) and tau proteins via cerebrospinal fluid (CSF) flux through astroglial aquaporin-4 (AQP4) water channels [104]. Functionally analogous to the peripheral lymphatic system, the glymphatic pathway is particularly active during sleep and is tightly regulated by arterial pulsatility, CSF dynamics, and astrocytic function. Impairments in glymphatic clearance have been associated with neurodegenerative diseases such as Alzheimer’s and Parkinson’s disease, as well as age-related cognitive decline [103,104]. While its function is primarily studied in relation to sleep, emerging evidence suggests that physical activity also plays a significant role in modulating glymphatic efficiency. In addition, recent studies have shown that different forms of physical training can improve sleep quality and even specific sleep stages, such as N3, which are closely linked to glymphatic clearance. For example, Von Holstein-Rathlou et al. reported that voluntary aerobic exercise improves deep-sleep–related glymphatic influx in awake, behaving mice [105].

Additionally, other animal studies have shed further light on the structural and functional determinants of glymphatic efficiency. Mestre et al. demonstrated that arterial pulsatility is not only associated with but actively drives cerebrospinal fluid flow along perivascular channels, and that reduced pulsatility, as occurs with aging or hypertension, markedly impairs solute clearance [106]. Complementing this, Smith et al. used two-photon imaging in live rodents to show that mislocalization of AQP4 channels from astrocytic end feet compromises glymphatic inflow and leads to greater retention of amyloid-beta in the interstitial space. These findings underscore the essential role of vascular dynamics and astrocytic polarity in maintaining effective brain clearance mechanisms [107].

### 5.2. Physical Activity as a Glymphatic Enhancer

Emerging preclinical studies suggest that regular physical activity can significantly enhance glymphatic transport. Von Holstein-Rathlou et al. demonstrated that mice subjected to voluntary wheel running exhibited increased interstitial solute clearance, associated with improved AQP4 polarization in astrocytic end-feet [108,109]. This polarization is critical for the convective influx of CSF into the brain parenchyma. Moreover, exercise was found to restore the glymphatic function of traumatic brain injury [110,111].

Exercise also indirectly promotes glymphatic efficiency by improving vascular health. Aerobic activity enhances arterial pulsatility and reduces cerebrovascular stiffness, two key drivers of perivascular CSF movement. Additionally, physical activity reduces chronic low-grade inflammation known to impair astrocytic AQP4 expression—thereby supporting the integrity of glymphatic architecture [112,113].

Additional experimental data reinforce the relationship between physical activity and glymphatic function across different contexts of brain health. For instance, Liu et al. (2013) demonstrated that moderate treadmill training in APP/PS1 transgenic mice enhanced AQP4 localization and glymphatic clearance, leading to a significant reduction in amyloid-beta deposition and improved memory performance [114]. Similarly, Reed et al. showed that chronic aerobic exercise not only improved perivascular CSF flow but also normalized vascular pulsatility and reduced blood–brain barrier permeability [115]. These findings suggest that exercise interventions may exert multifaceted benefits on the glymphatic pathway by restoring both astroglial and vascular components critical for efficient waste removal.

### 5.3. Molecular Mediators and Circadian Considerations

The link between exercise and glymphatic function appears to be mediated by multiple signaling pathways. Physical activity increases the expression of brain-derived neurotrophic factor (BDNF) and vascular endothelial growth factor (VEGF), both of which support vascular remodeling and astrocyte health [65,87,116]. Recent evidence also suggests that myokines such as irisin and METRNL may influence glymphatic dynamics via modulation of neuroinflammation and astrocytic plasticity [70,117].

Circadian rhythm, another regulator of glymphatic activity, may interact with exercise timing. Sleep–wake cycles modulate glymphatic clearance, and exercising at certain times of day could theoretically potentiate this effect. Although human data are limited, animal models indicate that exercising during the active phase (night in rodents) produces superior effects on CSF clearance [118].

Recent transcriptomic studies further support the link between exercise and glymphatic modulation via circadian mechanisms. For example, it has been found that wheel exercise training in aged mice altered the expression of core clock genes (e.g., *Bmal1*, *Per2*) in astrocytes, coinciding with improved AQP4 polarization and glymphatic influx [119,120]. Likewise, Thomas et al. demonstrated that timed exercise restored disrupted circadian rhythms in hippocampal astrocytes following sleep deprivation, suggesting that physical activity may resynchronize glial chronobiology and optimize clearance during subsequent sleep periods [121]. These findings point to a bidirectional interaction between exercise and circadian control of brain homeostasis, highlighting the need for chronobiological considerations in future interventions.

### 5.4. Implications for Neurodegenerative Disease and Healthy Aging

Enhancing glymphatic function through physical activity offers a promising, non-pharmacological strategy to reduce the cerebral accumulation of toxic proteins and prevent neurodegeneration. In Alzheimer’s disease models, physical activity has been shown to reduce Aβ burden and improve memory performance, potentially through glymphatic activation [122,123]. Furthermore, maintaining glymphatic efficiency may be especially relevant for aging populations, as both sleep architecture and CSF dynamics deteriorate with age.

In clinical contexts, integrating aerobic exercise programs particularly those that improve vascular compliance and sleep quality could be a valuable adjunct to current therapeutic strategies aimed at slowing cognitive decline [124,125]. Future research should aim to elucidate the optimal type, intensity, and timing of physical activity to maximize glymphatic clearance in humans.

Moreover, personalized interventions that align exercise routines with individual circadian profiles may further enhance glymphatic function and cognitive outcomes. Understanding these interactions can inform the design of chronotherapeutic exercise protocols, particularly for older adults at elevated risk for neurodegenerative diseases. Ultimately, targeting the glymphatic system through regular physical activity may represent a valuable element within a comprehensive strategy to support healthy brain aging and potentially reduce the risk of dementia.

## 6. Impact of Exercise on Functional Brain Network Connectivity in Older Adults

Physical exercise substantially and positively impacts functional brain network connectivity in healthy older adults, influencing multiple aspects of cognitive ability and brain function [124]. Studies show that acute bouts of exercise [125] and chronic exercise training enhance the structure and integration of large-scale brain networks. The outcomes are particularly noteworthy in maintaining cognitive resilience and reversing age-related decline [126]. Physical exercise has also been linked to increased connectivity across several major networks. The default mode network (DMN), active during rest and responsible for memory and self-referential thought, becomes more connected following aerobic exercise [127].

Greater connectivity of the DMN is related to better cognitive function, particularly in elderly individuals with superior executive functioning [128]. Similarly, improvement has been observed in the dorsal attention network (DAN), which is in charge of sustained attention, and in the frontoparietal network (FPN) and salience network (SAL), both of which play a pivotal role in attention, working memory, and cognitive control [129]. Another important contributor to exercise’s impact on the brain is that exercise can enhance network segregation, that is, functional differentiation among large-scale networks. Acute exercise, for instance, enhances segregation between SAL and DMN, and between SAL and the affect-reward network (also known as the reward network). This is opposite to the dedifferentiation with age that renders the edges between networks less discrete and is correlated with cognitive impairment [130]. Longitudinal analysis also yields evidence that chronic physical exercise improves connectivity between subcortical and frontal-subcortical networks, potentially affecting long-term brain network trajectories [131]. Older individuals experiencing normal cognition as well as those with mild cognitive impairment exhibit these effects, indicating that exercise may be a generally applicable brain-health intervention [132]. The benefits do not lie within a single network or cognitive process but are more of a general enhancement of the efficiency and integration of brain networks. Cognitive benefits related to exercise-induced changes in FBNC include executive function, memory, and overall cognitive enhancement. Direct associations between cognitive or physical benefits and FBNC changes have been tenuous, yet there is consensus in favor of exercise as a contributing factor to enhanced brain function [124]. Mechanisms thought to be behind these effects are increased neuroplasticity, neurogenesis, and improved neurovascular health [133].

In particular, length and intensity are of relevance. Increasingly longer interventions, such as 12 months of aerobic exercise, induce progressively larger effects on connectivity and cognition in the network [134]. However, even single bouts of moderate-to-vigorous exertion provide quantifiable enhancements in brain network organization [135].

Overall, regular physical exercise, especially of an aerobic nature, is significant in promoting functional brain network connectivity in older adults. Such changes are associated with enhanced cognitive ability and may offer neuroprotection against age-related cognitive impairment and neurodegenerative disease, validating the implication of physical activity in healthy brain aging [136].

## 7. Multicomponent Training and Cognitive Reserve: Toward Personalized Interventions

**Figure 1:**
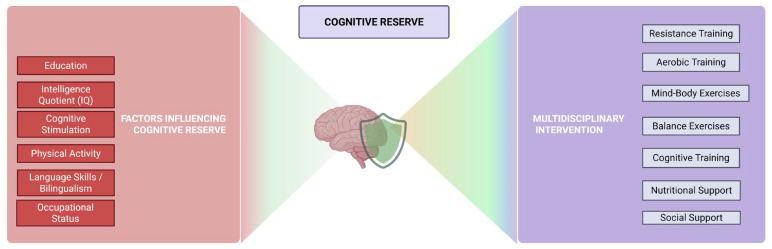
Multicomponent Training and Cognitive Reserve: Pathways Toward Personalized Cognitive Health.

Cognitive reserve is understood as the result of certain life experiences, along with genetic factors, that determine the variability in cognitive decline during adulthood, particularly in how the brain copes with aging. A higher level of cognitive reserve is associated with a delay in cognitive impairment and with better cognitive performance prior to decline (Figure 1) [137].

In other words, cognitive reserve is the brain’s ability to withstand neurological pathologies or disorders before showing symptoms of impairment [138]. Cognitive reserve acts as a protective factor against brain injuries and atrophy, and it reduces the effects-at the brain level-associated with aging and pathological damage [139].

Cognitive reserve cannot be directly measured or observed, so it must be assessed through other indirect indicators such as IQ, occupational status, physical activity, cognitive stimulation, language skills, etc. The mechanisms underlying the delayed onset of cognitive decline symptoms in individuals with higher cognitive reserve are not yet fully understood. However, certain factors that influence the capacity of this reserve are known, such as education, intelligence, and physical activity [138,139]. Multicomponent training includes various exercises and techniques aimed at developing physical practice. It typically incorporates strength, aerobic, resistance, and balance exercises, which gradually increase in intensity, duration and complexity. Previous studies have shown that multicomponent training, combined with other factors such as nutritional intervention, social support, and cognitive training, is effective and valid for individuals with cognitive impairment. Regarding physical practice, it is advisable to personalize and adapt it according to the individual’s physical abilities and functional level [140].

Longitudinal studies with older adults have shown that engaging in physical exercise at least three times a week reduces the risk of developing dementia in later years [139]. Physical activity promotes an increased release of brain-derived neurotrophic factor (BDNF), which is associated with delayed cognitive decline and the prevention of the accumulation of a substance related to the development of Alzheimer’s disease [141]. Previous research examining the relationship between physical activity and cognitive impairment has observed that individuals who engaged in a high level of physical exercise during their youth experience a delayed onset of cognitive impairment compared to those who did not exercise [142].

It has been shown that different types of exercise produce distinct cognitive effects. For example, resistance training is associated with improvements in executive functions and reasoning, mind–body exercises (such as tai chi) improve processing speed and attentional capacity, and other movement-based exercises enhance memory, processing speed and executive function [140]. Other studies have found that aerobic exercise can increase the volume of brain areas associated with cognitive aging, and that exercise can protect the hippocampus and prevent a decline in its cognitive functionality [142].

Interventions that combine physical exercise with cognitive training in a multitasking format have been shown to be more effective than single-modality exercise in improving cognitive function, and indirectly, cognitive reserve. Several studies have confirmed the benefits of combining cognitive training with physical exercise compared to a single intervention, both in neurophysiological terms (neuroplasticity and increased cerebral blood flow) and functional outcomes (physical and cognitive performance) [143].

Further research is still needed in this area, as there is limited evidence on the effects of multicomponent training on cognitive reserve. However, there is ample data supporting the benefits of both physical and cognitive training, at the level of both brain structures and cognitive functions. Combining an intervention that includes various types of physical activity (endurance, strength, balance, etc.) along with cognitive training could help slow the cognitive decline experienced by older adults, thereby promoting healthy and functional aging [138]. Furthermore, individualized interventions could be designed, tailored to each person’s needs, and implemented by a multidisciplinary team (nurse, psychologist, physiotherapist, etc.) [143].

## 8. Interaction Between Physical Activity and Circadian Rhythms in Cognitive Performance

There is substantial evidence regarding the effects of physical activity on cognitive performance. It has been observed that engaging in moderate-intensity exercise, even for short time periods, can enhance cognitive function [144]. Additionally, physical activity serves as a protective factor against the development of dementia, with evidence associating regular physical exercise to lower accumulation of Alzheimer’s disease biomarkers. In a longitudinal study conducted with an adult English population, participants who engaged in higher levels of physical activity demonstrated better cognitive performance—specifically in memory and verbal fluency tests.

Other findings suggest that acute physical exercise improves reaction time in male athletes and young sports students, while moderate-intensity exercise enhances attention in young female physical education students [144]. Moreover, research involving adults with depression has shown that physical activity is associated with improvements in working memory, inhibitory control, processing speed, attention, verbal fluency, and cognitive flexibility [145].

These improvements are explained by the neurophysiological changes induced by physical exercise, such as increased cerebral blood flow, which enhances the availability of oxygen and nutrients, as well as elevated concentrations of specific neurotransmitters and hormones [146]. Physical exercise enhances cognitive performance through the action of certain neurochemicals, such as insulin-like growth factor (IGF) and BDNF, or through increased hippocampal plasticity, which translates into improved memory capacity [147].

Circadian rhythms play a fundamental role in an individual’s physiological, cognitive, and behavioural functioning, causing fluctuations in hormone levels and various physiological processes, typically over a 24 h period. Physiological functions such as body temperature, sleep, appetite, and the secretion of melatonin and cortisol follow a circadian pattern regulated by the suprachiasmatic nucleus of the hypothalamus, which is synchronized by light–dark cues [148].

There are interindividual differences in how people adjust their internal clocks to environmental conditions, resulting in personalized circadian rhythms and what are known as chronotypes. Individuals can be categorized as “owls,” who perform better in the evening, or “larks,” who function better in the morning [149]. The existence of chronotypes is partly a matter of personal preference but is also associated with physiological variations. For example, larks tend to show earlier peaks in cortisol and melatonin levels, leading to earlier activity–sleep cycles and a peak in daily core body temperature in the early afternoon [150]. In this regard, a positive relationship has been observed between morning chronotypes and academic performance, with these individuals obtaining better results in primary mental abilities and higher average grades. Similarly, studies with German adolescents have found that those who preferred evening activities tended to show poorer academic performance [151].

Variations in circadian rhythms can influence neural functions such as cerebral perfusion, nerve conduction velocity, and the release of neurotrophic factors. These relationships underpin the association between circadian fluctuations, physiological functioning, and cognitive performance. However, determining the precise influence of circadian rhythms on cognitive performance is complex, as it requires accounting for factors such as accumulated fatigue and sleep-related disturbances. In any case, a review conducted by Xu et al. (2021) compiled findings from previous studies examining how certain cognitive functions are influenced by circadian rhythms [152]. For instance, working memory capacity appears to peak around midday and is associated with circadian markers such as metabolic activity, increased core body temperature, and elevated salivary cortisol levels. Other higher-order cognitive functions are also subject to circadian variation. Inhibitory control tends to peak in the late afternoon and is at its lowest early in the morning; cognitive flexibility shows poorer performance in the morning as well.

In individuals with disrupted circadian rhythms—such as night-shift workers—dysfunctions have been observed in sustained attention, executive functions, visuomotor performance, and processing speed [153].

The effects of physical exercise depend, among other factors, on the time of day at which the activity is performed. Circadian rhythms are also responsible for the variation and regulation of muscle strength, force production, stability, and muscle activation. Factors that explain the relationship between circadian rhythms and muscular performance include fluctuations in core body temperature and hormonal concentrations—such as insulin, cortisol, and testosterone—throughout the day [154].

Intense muscular activity leads to activation of the motor cortex and the prefrontal cortex, the latter of which is involved in executive functions. As circadian rhythms influence neurotransmitter release, the functioning of the prefrontal cortex may vary across the day. These observations support the relationship between physical exercise, cognitive performance, and circadian rhythms—suggesting that cognitive performance indicators may be affected by intense physical activity depending on the time of day it is performed.

Alertness levels tend to peak in the afternoon, improving reaction times, which coincides with increased muscular strength. During this time, perceived exertion and fatigue are reduced, while tolerance to maximal effort increases.

Furthermore, the activation of motor units during muscle contraction and the contractile properties of muscle fibres have been shown to vary throughout the day, with both strength and physical performance peaking in the afternoon. Prior studies have also found that this variability may differ by sex, as men tend to exhibit greater circadian variability in muscular strength compared to women [155].

Understanding the relationship between circadian rhythms and physical activity, as well as their interaction with cognitive performance, enables more effective planning of training and recovery programs. Specifically, identifying individuals’ chronotypes (through validated assessment tools) can help optimize performance based on the time of day.

In sports settings, this information is particularly relevant, as knowing an athlete’s chronotype and hormonal profile would allow for the personalization of training programs to maximize performance. Although implementing such strategies can be challenging—especially in team sports—it would be highly beneficial to consider these variables when aiming to optimize athletic outcomes [156].

Similarly, understanding the link between physical activity and cognitive performance, particularly how this relationship fluctuates throughout the day, may have practical applications in educational contexts. For instance, this knowledge could inform the design of school timetables to enhance academic performance.

## 9. Exercise Effects in Genetically At-Risk Populations (e.g., APOE **ε**4 Carriers)

### 9.1. Background: APOE ε4 and Alzheimer’s Disease Pathogenesis

The *Apolipoprotein E* (APOE) gene, located on chromosome 19q13.2, encodes a 299-amino acid protein responsible for lipid transport and injury repair in the central nervous system (CNS). Of its three isoforms—ε2, ε3, and ε4—the ε4 allele is the most significant genetic risk factor for sporadic late-onset Alzheimer’s disease (LOAD), present in approximately 20–25% of the general population and over 50% of AD cases [157].

Carriage of one ε4 allele increases AD risk approximately threefold, while homozygosity can increase lifetime risk by up to 12-fold and reduce the age of onset by nearly a decade [3]. Pathophysiologically, *APOE* ε4 is associated with several deleterious CNS effects: impaired Aβ clearance and aggregation, neuroinflammation, blood–brain barrier (BBB) dysfunction, synaptic loss, mitochondrial dysregulation, and reduced neurogenesis [158,159].

While *APOE* genotype is non-modifiable, lifestyle factors such as physical exercise have emerged as powerful modulators of cognitive health. Recent evidence suggests that exercise may not only slow cognitive decline generally, but interact favorably with the ε4 genotype to mitigate the penetrance of this genetic risk.

### 9.2. Genotype-Exercise Interactions: Epidemiological and Cognitive Evidence

A large body of observational and longitudinal cohort studies supports the idea that physical activity can differentially benefit ε4 carriers. In one of the earliest pivotal studies from the Cardiovascular Health Cognition Study, Laurin et al. found that elderly ε4 carriers engaging in regular physical activity had a substantially reduced risk of dementia compared to physically inactive counterparts, while non-carriers did not show such a pronounced association [160,161].

This effect has since been replicated across diverse populations. A prospective study by Smith et al. involving over 1200 cognitively healthy elders demonstrated that moderate-to-vigorous physical activity (MVPA) was linked to better memory and executive performance only in APOE ε4 carriers, and was not significant among ε3 homozygotes [161].

Notably, longitudinal data from the Framingham Heart Study also supports this: over a 10-year follow-up, higher levels of physical activity were associated with greater preservation of hippocampal volume in ε4 carriers but not in non-carriers [9]. These findings suggest a genotype-specific vulnerability that renders ε4 carriers more responsive to the neuroprotective effects of exercise [162].

### 9.3. Brain Imaging and Functional Connectivity: Evidence from Human Trials

Advanced neuroimaging modalities have become essential tools for elucidating how exercise influences brain architecture and network dynamics in individuals with genetic risk for Alzheimer’s disease. In *APOE* ε4 carriers, specific neuroanatomical regions and functional circuits show heightened sensitivity to pathological aging. Importantly, these same systems appear to exhibit a remarkable capacity for exercise-induced modulation, even in the absence of overt cognitive decline.

In the randomized controlled trial conducted by Lloyd et al., functional MRI was employed to assess changes in resting-state connectivity following a 6-month aerobic exercise intervention. In ε4 carriers, the intervention led to increased intra-network connectivity within the default mode network (DMN), particularly in the posterior cingulate cortex and medial prefrontal cortex. These regions are integral to episodic memory retrieval and are among the earliest to show metabolic decline in preclinical AD. The restoration of DMN integrity suggests a selective enhancement of functional network efficiency in genetically susceptible individuals [163].

Structural adaptations have also been captured through high-resolution anatomical MRI. The study by ten Brinke et al. using voxel-based morphometry revealed that ε4 carriers with higher aerobic fitness had more robust grey matter volume in medial temporal regions, including the anterior hippocampus and entorhinal cortex. These areas are critical for relational memory encoding and spatial navigation. The volumetric preservation was found to scale with aerobic capacity, suggesting that cardiovascular efficiency may directly influence regional brain plasticity in this high-risk group [164].

White matter integrity, a key substrate of inter-regional communication, has also been implicated. Data from the ADNI cohort showed that ε4 carriers with superior baseline cardiorespiratory fitness demonstrated attenuated accumulation of white matter hyperintensities (WMHs) and lower rates of periventricular lesion expansion over a two-year follow-up. These findings are notable given that WMHs are predictive of executive dysfunction, gait disturbances, and increased risk of conversion from MCI to dementia. The preservation of white matter pathways in physically active ε4 carriers suggests that exercise may help maintain global neural connectivity and prevent microvascular compromise, which is particularly relevant in aging brains with genetic vulnerability [165].

Furthermore, diffusion tensor imaging (DTI) analyses nested within these datasets have identified higher fractional anisotropy and lower mean diffusivity in physically active ε4 carriers, pointing toward improved microstructural organization in tracts such as the cingulum bundle and uncinate fasciculus—tracts linking frontal and temporal lobes and supporting memory and emotion regulation. These changes imply that exercise may enhance axonal integrity and myelin maintenance, providing a mechanistic substrate for cognitive resilience in the face of elevated Alzheimer’s risk.

### 9.4. Neurobiological Mechanisms: BDNF, Inflammation, Amyloid Clearance

The mechanistic basis for these genotype-specific effects lies in the neurobiological profile of *APOE* ε4 carriers, which is characterized by enhanced oxidative stress, reduced synaptic plasticity, and impaired proteostasis.

Exercise-induced brain-derived neurotrophic factor (BDNF) has been identified as a key mediator of neurogenesis and synaptic integrity. Interestingly, ε4 carriers tend to have lower basal BDNF levels, but exhibit greater upregulation of BDNF in response to aerobic training compared to non-carriers, as demonstrated in recent peripheral and CSF studies [166,167].

Additionally, aerobic activity reduces cerebral pro-inflammatory cytokines (e.g., IL-6, TNF-α) and enhances microglial phenotype polarization toward anti-inflammatory profiles, which is particularly relevant in the context of ε4-driven neuroinflammation [168].

In preclinical transgenic models expressing human ε4 alleles, treadmill exercise has been shown to reduce amyloid-β deposition via enhanced expression of neprilysin and insulin-degrading enzyme, and by promoting glymphatic clearance mechanisms key processes in early AD pathology [169].

### 9.5. Epigenetics and Genetic Regulation: Exercise as a Modulator in APOE ε4 Carriers

Beyond immediate neurochemical shifts, physical activity acts as an epigenetic modulator, capable of influencing gene expression without altering DNA sequence—an especially relevant mechanism in genetically at-risk individuals.

Recent studies in both humans and murine ε4 models indicate that exercise can induce histone acetylation and DNA demethylation in brain regions such as the hippocampus, thereby increasing the expression of genes linked to synaptic plasticity, anti-apoptotic signaling, and mitochondrial biogenesis [170]. In ε4 carriers whose neurons show early vulnerability to epigenetic silencing of neuroprotective genes this modulation can shift the trajectory away from neurodegeneration.

In particular, microRNAs (miRNAs) small non-coding RNAs that regulate post-transcriptional gene expression—are emerging as key players. For example, miR-132 and miR-206, both downregulated in ε4-associated AD brains, have been shown to be upregulated by aerobic and resistance training, restoring BDNF and synaptophysin signaling [171].

This epigenetic influence extends to immune regulation, where exercise has been shown to decrease expression of miRNAs associated with neuroinflammation (e.g., miR-155), offering a potential mechanism for the attenuation of the APOE ε4-induced inflammatory phenotype [172].

### 9.6. Clinical Trials and Personalized Interventions

Several randomized controlled trials (RCTs) and large-scale longitudinal studies have stratified participants by *APOE* genotype to assess exercise response. One of the most comprehensive, the AIBL Active Trial, is evaluating the effects of a 24-month aerobic training program on amyloid load (via PET imaging), hippocampal volume, and cognition in ε4 carriers versus non-carriers. Preliminary results suggest enhanced amyloid clearance and delayed cognitive decline in physically active ε4 carriers [173].

Similarly, the FINGER study—a landmark multidomain intervention trial—found that APOE ε4 carriers benefitted significantly from structured physical activity combined with diet, cognitive training, and vascular risk control. Notably, ε4 carriers exhibited equal or greater gains in executive function and processing speed compared to non-carriers, suggesting that genetic risk does not preclude cognitive plasticity [174].

Emerging protocols like the MAPT (Multidomain Alzheimer Preventive Trial) and the PreDIVA trial also support the idea that tailored interventions based on genetic risk profiles may yield superior outcomes when compared to uniform public health recommendations.

Moreover, several neuroimaging-focused substudies are evaluating the impact of HIIT (high-intensity interval training) versus moderate-intensity continuous training in ε4 carriers, aiming to determine the optimal exercise prescription for neuroprotection in this high-risk group.

### 9.7. Sex Differences and Hormonal Interactions in APOE ε4 Responses

There is increasing recognition that sex interacts with APOE genotype and modulates the neural response to exercise. Epidemiological data show that female ε4 carriers have a higher lifetime risk of AD than males with the same genotype, potentially due to hormonal interactions (e.g., estrogen decline post-menopause) and differential lipid metabolism [175].

Recent analyses from the Mayo Clinic Study of Aging revealed that physically active female ε4 carriers demonstrated greater preservation of hippocampal volume and verbal memory compared to physically inactive counterparts, whereas the same was not statistically significant in male carriers [176].

Moreover, animal models have shown that estrogen replacement therapy combined with exercise synergistically restores synaptic protein expression and reduces amyloid burden in female ε4 knock-in mice [177], suggesting a sex-specific window of neuroplasticity that could be harnessed clinically.

These findings underscore the importance of sex- and genotype-tailored exercise protocols in preventive neurology, especially considering the disproportionate burden of Alzheimer’s disease in aging women.

### 9.8. Precision Prevention: Toward Genotype-Guided Cognitive Health Strategies

The convergence of genetics, lifestyle science, and digital health is giving rise to a precision prevention model, in which *APOE* status is used not only for risk stratification but also to personalize behavioral interventions, especially exercise regimens.

Digital phenotyping tools, including wearable devices (e.g., heart rate variability, sleep quality, and physical activity monitors), now allow for continuous, real-world tracking of lifestyle adherence and physiological response. These tools can inform adaptive feedback loops in which ε4 carriers receive real-time adjustments in exercise intensity, duration, or recovery strategies to optimize neurocognitive outcomes.

Furthermore, integration of *APOE* status with other biomarkers (e.g., CSF tau/Aβ, neurofilament light chain, or plasma GFAP) enables early identification of individuals in preclinical or prodromal stages, who may benefit most from high-intensity or multimodal training programs [178].

In this context, exercise is not merely a behavioral recommendation but a biological intervention, capable of reshaping the trajectory of brain aging—even in those with high genetic risk.

## 10. Physical Activity and the Prevention of Mild Cognitive Impairment (MCI) Progression

Mild Cognitive Impairment (MCI) represents a clinically significant transitional state between normative aging and dementia, particularly Alzheimer’s disease (AD). Characterized by measurable cognitive decline without substantial impairment in daily functioning, MCI affects approximately 15–20% of adults aged 65 and older [179]. Alarmingly, conversion rates to dementia among MCI patients are estimated at 10–15% per year, making early intervention critical [180].

Among available non-pharmacological strategies, physical activity has emerged as the most consistently supported behavioral intervention for mitigating MCI progression. Its neuroprotective effects span structural, metabolic, and molecular domains, offering both symptomatic relief and potential disease-modifying benefits.

### 10.1. Neurocognitive Outcomes of Exercise in MCI Populations

Multiple randomized controlled trials (RCTs) have demonstrated that structured physical activity significantly attenuates cognitive decline in individuals with MCI. The EXERT trial, a large-scale, 18-month Phase 3 study, evaluated moderate-intensity aerobic exercise versus stretching control in physically inactive older adults with amnestic MCI. Participants in the aerobic group showed greater stability in executive function and episodic memory, with neuroimaging indicating preservation of hippocampal volume and cortical thickness [181,182]

Likewise, Maillot et al. evaluated the potential of exergame training based on physically simulated sport play as a form of physical activity with cognitive benefits for older adults. Using a pretest-training-posttest design, they compared an experimental group (24 × 1 h sessions) with a no-treatment control group. Results showed that participants in the training group improved significantly in physical function and in cognitive measures of executive control and processing speed, though not in visuospatial abilities. The findings suggest that exergames may offer an attractive and effective way to promote lifestyle changes and enhance functional skills relevant to daily living in older adults [183].

Interestingly, benefits are not restricted to aerobic training. Resistance exercise has been shown to enhance executive function, working memory, and task switching. The SMART trial found that biweekly progressive resistance training over 6 months improved the Alzheimer’s Disease Assessment Scale-Cognition (ADAS-Cog) scores in MCI patients and was associated with functional connectivity changes in the posterior cingulate cortex, a region highly vulnerable in AD [184].

### 10.2. Brain Imaging Biomarkers: Structural and Functional Preservation

Advancements in neuroimaging have provided critical insights into how physical activity influences brain structure and function in older adults with Mild Cognitive Impairment (MCI), a population at elevated risk for progression to dementia. Among the most consistent findings is the association between sustained aerobic fitness and maintenance of medial temporal lobe integrity, particularly in the hippocampus and entorhinal cortex—regions known to undergo early and progressive atrophy in MCI [185].

In a longitudinal study conducted by Erickson et al., older adults diagnosed with MCI who engaged in regular aerobic activity demonstrated attenuated hippocampal volume loss over a 12-month period, as measured by structural MRI. Notably, volume preservation was not limited to gross anatomy but involved subfields implicated in memory encoding, such as the dentate gyrus and CA1 region, suggesting a region-specific neuroprotective effect associated with cardiorespiratory fitness [186].

Beyond structural measures, functional MRI studies have reported that physical activity modulates task-evoked activation in frontoparietal control networks, which support executive function and attentional regulation domains typically impaired early in MCI. For example, older adults engaging in structured physical activity exhibit enhanced blood-oxygen-level-dependent (BOLD) responses in the dorsolateral prefrontal cortex and inferior parietal lobule during working memory tasks. This increase in neural efficiency appears to compensate for declining medial temporal lobe function, reflecting a shift toward prefrontal recruitment in cognitively at-risk populations [187].

Molecular imaging modalities, particularly FDG-PET, have further highlighted the metabolic effects of physical activity on the aging brain. MCI participants with higher habitual physical activity levels show preserved cerebral glucose metabolism in key association cortices—most notably the posterior cingulate and precuneus, areas that typically exhibit early hypometabolism in Alzheimer’s disease. Additionally, recent evidence suggests that long-term aerobic training may reduce fibrillar amyloid deposition, as measured by PET tracers such as florbetapir, indicating a potential for physical activity to modify disease trajectory at the neuropathological level [187,188].

A recent single-blind randomized controlled trial investigated the efficacy, safety, and potential mechanisms of a multimodal intervention designed to enhance cognitive function in individuals with mild cognitive impairment (MCI) living in the community. Unlike previous research conducted mainly in high-income countries, this study included 120 participants with MCI who were randomly assigned to either a multimodal intervention group or a control group receiving regular health education. After 12 weeks, the intervention group showed significant improvements in global cognition (Mini-Mental State Exam [MMSE] total score), as well as in domains such as recall, language, memory, processing speed, and executive function, compared to controls. Neuroimaging data further revealed enhanced structural and functional connectivity associated with language, concentration, executive functioning, memory, and recall. These findings support the potential of multimodal approaches to improve cognitive performance and brain network integrity in individuals with MCI [189].

Emerging analyses also suggest that training-induced structural resilience may not be uniformly distributed across individuals but may depend on baseline brain reserve and vascular burden. Subgroup analyses indicate that individuals with higher white matter hyperintensity load at baseline may derive greater structural benefits from exercise, potentially due to improved perfusion and endothelial function elicited by physical activity.

### 10.3. Molecular and Cellular Effects: Neuroinflammation, Insulin Resistance, and Mitochondrial Function

At the cellular level, MCI is increasingly recognized as a state of heightened neuroinflammatory activity, metabolic inflexibility, and synaptic vulnerability. Exercise counteracts these processes through multiple converging pathways.

Chronic physical activity reduces circulating and CNS levels of pro-inflammatory cytokines (e.g., IL-6, TNF-α), improves mitochondrial efficiency, and enhances insulin signaling in the brain—a critical factor as MCI is strongly associated with brain insulin resistance and “type 3 diabetes” mechanisms [190,191]. Resistance and aerobic training have both been shown to upregulate insulin receptor expression and glucose transporter-4 (GLUT4) in the hippocampus, improving glucose utilization in MCI brains.

Furthermore, exercise increases neurotrophic factors such as BDNF and IGF-1, promoting neurogenesis and synaptic remodeling. BDNF levels are often depleted in MCI patients, and multiple studies have confirmed that exercise-induced increases in BDNF correlate with improved cognitive trajectories [192].

Mitochondrial biogenesis is another target: endurance training activates PGC-1α pathways, which drive mitochondrial replication and antioxidant defenses—offering resilience against the oxidative damage common in MCI [191].

### 10.4. Psychosocial and Functional Impacts

In addition to direct neurobiological effects, physical activity confers indirect benefits that may influence MCI outcomes. Structured exercise improves sleep quality, mood, vascular health, and physical function, all of which are tightly linked to cognitive trajectories [193].

Depressive symptoms are common in MCI and significantly increase risk of progression. Meta-analyses show that aerobic and multimodal training can reduce depression scores by 20–30%, and this correlates with improvements in attention and working memory [194].

Functionally, exercise enhances gait speed, balance, and dual-task performance, mitigating fall risk and preserving autonomy critical for maintaining independence and delaying institutionalization.

### 10.5. Multimodal and Personalized Approaches in MCI Prevention

Recent trials have embraced multicomponent intervention strategies to maximize the protective potential of physical activity. The FINGER trial, which included individuals with MCI or high dementia risk, combined physical training with nutritional counseling, cognitive stimulation, and vascular risk management. Results showed significant improvements in executive function, processing speed, and global cognition [195].

Similarly, the MAPT trial evaluated the effects of omega-3 supplementation and physical activity in older adults with subjective cognitive decline and MCI. Although overall effects were modest, greater adherence to physical activity protocols was independently associated with reduced hippocampal shrinkage and better verbal fluency [196].

These findings underscore the importance of individualizing interventions based on baseline fitness, comorbidities, and cognitive phenotype. Wearable technologies and remote monitoring platforms are now facilitating adaptive, home-based programs that maintain long-term engagement and enable stratified interventions based on objective data [197].

### 10.6. Early Identification and Public Health Implications

Given the global demographic shift toward aging populations, strategies to delay or prevent dementia onset at the MCI stage represent a critical public health priority. Physical activity stands out not only as a scientifically validated neuroprotective tool but also as a scalable, low-cost intervention with minimal adverse effects and broad systemic benefits.

Early identification of individuals with MCI particularly through community-based cognitive screening, wearable digital biomarkers, and predictive algorithms using electronic health data—enables the timely deployment of physical activity interventions before irreversible neurodegeneration sets in. Importantly, recent findings indicate that even low-intensity physical activity (e.g., brisk walking 30 min daily) can yield cognitive and structural brain benefits in preclinical stages [198,199].

The WHO and the Lancet Commission on Dementia Prevention have now both included physical activity among the top modifiable risk factors for dementia, underscoring the need for national and regional policies that promote active aging environments from urban design to workplace ergonomics and social support networks [200].

Furthermore, primary care providers are increasingly urged to incorporate “exercise prescriptions” into clinical management plans for patients with MCI, similar to protocols for hypertension or diabetes. Initiatives such as the “Exercise is Medicine” program and the American College of Sports Medicine’s Brain Health Guidelines are helping to operationalize this shift in preventive neurology. In underserved populations—where access to cognitive rehabilitation or pharmacological interventions may be limited—physical activity programs represent a uniquely equitable strategy, capable of reducing socioeconomic disparities in cognitive aging outcomes [201,202].

## 11. Exercise as an Epigenetic Modulator in Brain Aging

**Figure 2:**
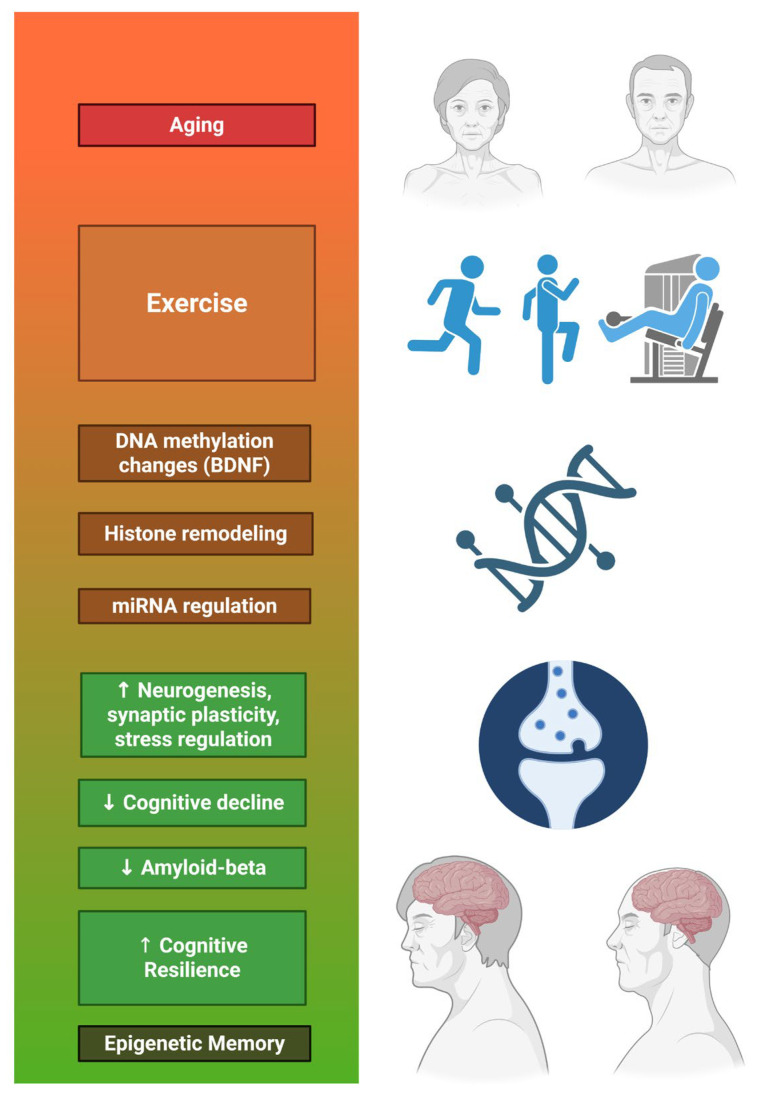
From Exercise to Epigenetic Memory.

Aging is characterized by progressive deterioration in molecular and cellular mechanisms resulting in loss of cognition and increased vulnerability to neurodegenerative disorders [203], such as Alzheimer’s disease [204]. Among the biological indicators of aging, epigenetic alterations, specifically modifications of DNA methylation [205], histone marks [206], and non-coding RNA expression [207], are becoming increasingly recognized as central to explaining age-dependent brain dysfunction (Figure 2). Physical exercise, on the other hand, is a strong epigenetic modulator of brain aging, influencing cognitive functioning and brain plasticity through complex and multi-factorial molecular mechanisms [36,168]. In this sense, exercise interventions have shown significant promise to reverse or impede age-associated cognitive impairments and general brain health [208]. The overview of Zheng et al. (2025) clearly states that, at the epigenetic level, exercise induces changes that dictate gene expression patterns crucial for memory, neuroplasticity, and stress resilience, highlighting the need for individualized exercise interventions [209]. Specifically, exercise can modify DNA methylation patterns, including reversal of age-associated epigenetic drift [210]. A basic example is the exercise-induced demethylation of the promoter of the brain-derived neurotrophic factor (BDNF) gene, leading to higher expression of BDNF, which is a key neurotrophin involved in synaptic plasticity and memory [211]. Exercise can also modify the activity of DNA methyltransferases in the hippocampus, leading to better synaptic function and cognitive resilience [212]. Alterations in histone acetylation and methylation states have also been reported. These can result in chromatin remodeling and gene transcription, as increased hippocampal H4K12 acetylation levels of aged animals, following a 2-week treadmill exercise, associated with the reestablishment of young gene expression patterns and enhanced cognitive function [213]. Such histone modifications enable increased access to transcription for genes that facilitate neurogenesis and plasticity, offering significant benefits to promote a healthy brain while aging [214,215,216].

MicroRNAs, a class of non-coding RNAs with central regulatory functions, are also epigenetically controlled by exercise. In this sense, exercise reprograms the expression patterns of hippocampal miRNAs, influencing neurogenesis, memory consolidation [217], and stress coping [218]. These changes may underlie the observed effects of exercise on cognitive aging and neurological stressor [208]. Apart from regulating epigenetic mechanisms, exercise enhances neuroprotection and reverses pathological mechanisms that define neurodegenerative diseases [165,171]. As an example, BDNF signaling pathway upregulation through epigenetic regulation has been noted to reduce amyloid-beta accumulation and improve cognitive function in models of Alzheimer’s disease [171,219]. Besides direct gene regulation, exercise also triggers more widespread bioenergetic and transcriptional adaptations that enhance the resistance of the brain to metabolic, oxidative, and proteotoxic stress, which are considered important features of aging-related neuronal dysfunction [220]. Collectively, these adaptive processes, derived from the beneficial effects of regular exercise, although most current research focuses primarily on running-based interventions, might form part of the so-called “epigenetic memory” designed to sustain cognitive function and mental health [220].

## 12. Emerging Technologies: Tele-Exercise, Virtual Reality, and Cognitive-Motor Gamification

In recent years, the convergence of neuroscience, digital health, and gerontechnology has catalyzed a new era in cognitive intervention: one in which emerging technologies complement or even enhance traditional physical activity paradigms to optimize brain health in older adults. Tele-exercise platforms, immersive virtual reality (VR) environments [188], and gamified cognitive-motor training systems are transforming how physical activity is delivered, monitored, and experienced in populations at risk for cognitive decline. These innovations hold particular promise for mitigating sedentary behavior, improving adherence, and targeting multiple cognitive domains simultaneously through enriched sensorimotor engagement [221].

### 12.1. Tele-Exercise and Remote Monitoring: Accessibility, Adherence, and Cognitive Outcomes

Tele-exercise refers to the remote delivery of structured physical activity programs using digital communication tools—ranging from basic video conferencing to AI-driven adaptive coaching systems. Originally deployed to overcome barriers in rural and mobility-restricted populations, tele-exercise gained massive traction during the COVID-19 pandemic, and now represents a scalable, sustainable model for promoting long-term physical activity in cognitively vulnerable populations [222].

Recent clinical trials have shown that remotely supervised exercise programs can match or even surpass in-person formats in maintaining engagement and cognitive gains. For instance, the PROMOTE study demonstrated that a 12-month tele-exercise intervention in older adults with mild cognitive impairment (MCI) produced significant improvements in executive function, processing speed, and depressive symptoms, while also reducing fall risk and increasing cardiorespiratory fitness [223].

These effects are thought to be mediated by sustained physiological and psychological benefits: tele-exercise programs increase daily energy expenditure, improve metabolic and vascular parameters, and promote routine—all of which are associated with reduced hippocampal atrophy and enhanced prefrontal cortical activation [2]. Moreover, the integration of wearable devices (e.g., heart rate monitors, inertial sensors) and mobile applications enables continuous monitoring of compliance, exertion, and sleep, facilitating precision feedback loops and remote supervision by clinical staff or AI agents.

Additionally, machine learning algorithms are being trained to detect preclinical decline in gait, reaction time, and dual-task cost through video or sensor data, allowing platforms to adapt intensity or modality dynamically, and flagging early signs of frailty or cognitive deterioration [3].

Tele-exercise thus not only democratizes access to brain-healthy physical activity but may also evolve into a digital biomarker hub for cognitive risk stratification and preventive care.

### 12.2. Virtual Reality-Based Physical and Cognitive Training: Neuroplasticity in Immersive Environments

Immersive virtual reality (VR) offers a unique platform to simultaneously engage motor, cognitive, and emotional systems. Unlike traditional exercise, VR allows for precise control over sensory input, environmental complexity, and real-time feedback, facilitating highly personalized and adaptive training paradigms [224,225].

Neuroimaging studies have shown that older adults who engage in VR-enhanced exercise (e.g., cycling while navigating virtual environments or performing memory tasks) exhibit greater activation of the dorsolateral prefrontal cortex, parietal lobes, and hippocampus, as well as increased BDNF levels and improved spatial orientation. These regions are critical for executive control and episodic memory, and are among the earliest affected in Alzheimer’s disease.

A growing number of RCTs support the cognitive benefits of VR-based interventions. For example, a 6-week VR-assisted dual-task walking program led to significant improvements in attention, working memory, and gait variability among older adults with MCI compared to non-immersive training.

Moreover, the embodied cognition framework suggests that sensorimotor interaction with enriched environments facilitates learning and memory consolidation, particularly in aging brains where traditional declarative learning mechanisms may be impaired.

Importantly, the motivational and hedonic elements of VR (e.g., narrative context, novelty, immediate feedback) improve adherence and affective engagement, two major challenges in long-term physical activity programs for seniors.

VR training also has potential applications in post-stroke rehabilitation, Parkinson’s disease, and frailty prevention, indicating its versatility across neurodegenerative and geriatric syndromes. Ongoing research is exploring the integration of neurofeedback, haptic interfaces, and bioadaptive scenarios to further personalize the experience and amplify neurocognitive plasticity.

### 12.3. Cognitive-Motor Gamification: Enhancing Dual-Task Performance and Cognitive Reserve

Cognitive-motor gamification involves embedding cognitive challenges into physical tasks, creating dual-task paradigms that train executive function, divided attention, and visuospatial coordination under dynamic conditions. Unlike traditional cognitive training, these interventions engage neural networks in an ecologically valid and functional manner, reflecting real-world demands such as walking while talking or navigating while planning.

Meta-analyses show that dual-task interventions outperform both physical or cognitive training alone in enhancing global cognition, gait speed under cognitive load, and dual-task cost, especially in MCI and early dementia populations [226,227].

Notable examples are the exergame-based programs, which improved inhibitory control and fall risk in older adults after just 8 weeks of use. Neuroimaging revealed increased prefrontal cortex activation and better synchronization across task-relevant neural networks [228]. Gamified platforms such as Nintendo Wii Fit, Dance Dance Revolution, and more recently AI-driven systems like NeuroGym or XRHealth, incorporate adaptive difficulty, point systems, and narrative progression, which enhance motivation, reward processing, and behavioral adherence [229,230].

Moreover, gamified training stimulates dopaminergic pathways, implicated in motivation and learning, and may compensate for age-related decline in reward sensitivity, thus maintaining engagement even in apathy-prone populations. Hybrid platforms are now being tested in assisted living facilities and memory clinics, and initial data suggest that gamified dual-task training can enhance cognitive reserve, delay institutionalization, and improve quality of life in older adults with subjective cognitive decline or early-stage dementia [231].

## 13. Sex Differences in Neurocognitive Response to Exercise in Older Adults

Exercise has emerged as a preeminent non-pharmacological intervention for alleviating age-associated cognitive decline [232]. However, an accumulation of evidence demonstrates that cognitive benefits conferred by exercise are not universally distributed across the population [233], with biological sex emerging as a significant moderator of such effects in older adults [234]. A recent cross-sectional study suggests that older healthy women, in particular, exhibit profound improvement in executive functions such as planning, cognitive flexibility, and inhibition following exercise interventions [235]. These gains have been seen in a variety of exercise modality types, including aerobic training, resistance training, and multimodal training [236].

Regarding specific sex differences, women, relative to men, may see greater gains on executive function and visuospatial abilities, highlighting that specific cognitive domain responsive to exercise may differ by sex [237]. Additionally, a previous systematic review and meta-analysis indicated that men and women experience dissimilar improvements in cognitive performance following exercise, while the type of exercise intervention was considered a significant moderator [238]. The results from the recent study of Krumpolt et al. showed that women perform more slowly initially on certain reaction time tasks but exhibit larger improvements following exercise training. On the other hand, men may exhibit greater improvement in tasks of controlled and automatized processing, underlining the complexity of sex-differential trajectories in cognitive adaptation to physical exercise, in the specific case, combined fitness and recreational sports [239]. Several biological mechanisms have been implicated in explaining sex differences in cognitive response to exercise in older adults, including neuroplasticity [240]. Additionally, emphasis has been given on analyzing the impact of sex steroid hormones, such as estrogen and testosterone, which have well-defined effects on upregulating the hippocampal structure and the power of synaptic plasticity [241]. Ageing declines in these hormones, particularly in postmenopausal women, which might affect exercise-induced neurobiological processes in a sex-specific manner. For instance, estrogen is demonstrated to influence the expression of neurotrophic factors and modulate neurotransmitter systems involved in cognitive function [242]. These hormonal mechanisms may be the source of women’s higher susceptibility to exercise in executive function domains, as indicated by a study in female exercising animals [243]. Beyond hormone effects, neurotrophic factors such as BDNF are central to the mediation of exercise effects on cognition [189]. BDNF plays a role in synaptic plasticity, neuronal survival, and long-term potentiation, processes underlying learning and memory [244]. Studies suggest that older women are capable of significant increases in circulating BDNF levels with aerobic exercise, which could augment the exercise-induced cognitive improvements also demonstrating overall BDNF, compared to older men after a 30 min bout of aerobic exercise [233]. Additionally, long-term physical activity adherence has been associated with increased brain volume in regions responsible for executive function, such as the dorsolateral prefrontal cortex [223]. These changes in structure appear to be more pronounced in women, possibly due to interactions between activity levels, hormonal status, and neurotrophic support. Genetic and physiological processes may also underlie these observed sex differences. For instance, sex-specific expression of genes for neurodegenerative risk, such as the APOE-ε4 allele [175,245], may modify the efficacy of exercise on cognitive outcomes [224]. Similarly, individual differences in cardiovascular fitness, inflammatory markers, and metabolic functioning, each of which may affect the brain’s response to exercise, may interact with sex to impact cognitive aging trajectories [225]. Moreover, the exercise mode, intensity, and duration also appear to be significant moderators regarding the extent and nature of cognitive benefits between the sexes. While aerobic, resistance, and multimodal exercise are all associated with cognitive benefits, the relative efficacy of each modality may differ in men and women [238,246]. Adherence to public health guidelines (i.e., engaging in at least 150 min of moderate-intensity exercise per week) has been found in both sexes to be consistently related to healthy cognitive function [226,247]. Finally, a small but consistent advantage of males is highlighted in visual-spatial working memory; nevertheless, this advantage appears too small to fully explain the larger sex differences in spatial abilities, particularly in mental rotation, while women show a corresponding advantage in pure location memory tasks. Together, these findings emphasize the need for biological sex to be handled as a primary variable in exercise-based cognitive interventions in older adults. The interaction between sex-specific neurobiology, exercise modality, and cognitive domain suggests that one-size-fits-all might be an inefficient approach to optimizing brain health with physical exercise. Instead, there is now growing support for the development of personalized exercise prescriptions grounded in sex differences in hormonal status, brain structure, genetic risk, and neuroplastic potential. Such individualized approaches can enhance the efficacy of exercise interventions for the development of cognitive resilience and healthy brain aging [227,248,249].

## 14. Limitations and Future Applications

This narrative review has several limitations that should be acknowledged. First, although we aimed to provide a comprehensive synthesis, the heterogeneity of exercise protocols across studies including differences in type, intensity, duration, frequency, and supervision makes it difficult to derive universal recommendations. Second, as a narrative review, our approach does not follow systematic review procedures, and therefore may be influenced by selection bias despite our structured search strategy. Third, publication bias is a recognized issue in exercise–cognition research, as studies reporting positive results are more likely to be published, potentially inflating the apparent consistency of benefits. Finally, findings from animal models, while valuable for mechanistic insights, may not fully translate to humans and should be interpreted with caution. These studies are invaluable for identifying potential pathways and mediators such as BDNF, irisin, or glymphatic adaptations, yet their findings should be interpreted carefully when applied to humans. Differences in biological complexity, physiology, and environmental context mean that results in animal models should be regarded primarily as hypotheses that require further confirmation in clinical and translational research.

Future research should address these gaps with greater precision. Large-scale and long-term randomized controlled trials stratified by genetic risk factors such as APOE ε4 are needed to better understand inter-individual variability in response to exercise. The integration of precision medicine approaches is essential to personalize exercise prescriptions according to genetics, sex, circadian rhythms, and baseline cognitive status. In addition, more mechanistic human studies are required to clarify how molecular biomarkers including BDNF, irisin, and IGF-1 mediate cognitive benefits of physical activity. Together, these directions will help move the field toward more robust, personalized, and mechanistically grounded recommendations for cognitive health in aging.

In sum, as this work is based on a narrative review, it represents an interpretative synthesis of the available evidence rather than a systematic meta-analysis. The included studies vary in methodology, design, and population characteristics (both human and non-human), which limits the generalizability of some conclusions. Therefore, the statements and recommendations presented should be interpreted with caution and within the context of this heterogeneity.

## 15. Practical Applications

- **Clinical practice**: Exercise prescriptions should be incorporated into routine geriatric care, with aerobic and multicomponent programs tailored to cognitive and physical baselines.
- **Public health**: Community-based physical activity programs can reduce dementia incidence and promote healthy aging at the population level.
- **Personalized interventions**: Programs should consider individual variability—such as APOE ε4 status, sex, and chronotype—to maximize cognitive benefits.
- **Technology integration**: Tele-exercise, virtual reality, and gamified training offer innovative ways to increase adherence, especially in remote or mobility-limited populations.
- **Multidomain strategies**: Combining physical activity with cognitive training, nutrition, and social engagement enhances outcomes and may build cognitive reserve.
- **Policy implications**: Governments and institutions should support infrastructures and guidelines promoting active aging environments and lifelong physical activity habits.
- **Training load:** Exercise prescriptions should also define intensity and volume. Current recommendations suggest aerobic training at 60–75% of VO_2_max (or HRmax), with higher intensities (85–95% HRpeak) applied intermittently when feasible. For resistance training, loads of 70–80% of one-repetition maximum (1RM) are advised. Balancing intensity and safety is particularly important in older adults [250].

## 16. Conclusions

Physical exercise emerges as a powerful and accessible tool to preserve and enhance cognitive function in aging. Its neuroprotective effects are supported by a wide range of studies showing improvements in memory, executive function, and brain connectivity in both healthy older adults and those at cognitive risk. These benefits are mediated by systemic and molecular pathways, including neurotrophic signaling, improved vascular and glymphatic function, and modulation of inflammation and circadian rhythms. Furthermore, the interaction between physical activity and individual factors—such as genotype, sex, chronotype, and baseline fitness—highlights the need for personalized approaches. Integrating exercise with digital technologies and multicomponent interventions may optimize outcomes and facilitate broader implementation. Overall, promoting physical activity represents a critical, non-pharmacological strategy in the prevention of cognitive decline and dementia.

## Data Availability

No new data were created or analyzed in this study.
